# Group Means and Inter-Individual Analysis in Post-exercise Hypotension: Effects of Citrulline Malate Oral Supplementation

**DOI:** 10.5935/abc.20190152

**Published:** 2019-08

**Authors:** Leandro C. Brito

**Affiliations:** Universidade de São Paulo - Escola de Educação Física e Esporte, São Paulo, SP - Brazil

**Keywords:** Cardiovascular Diseases, Blood Pressure, Hypertension, Mortality & Morbidity, Exercise, Post-Exercise Hypotension, Citrulline

Hypertension is pointed out as one of the most aggressive risk factor for cardiovascular morbidity and mortality, since it is directly associated with nearly 8 million obits per year related to cardiovascular diseases such as cardiac arrest or stroke.^1^ Hypertensives with low levels of physical activity present higher risk of cardiovascular morbidity and mortality even when receiving anti-hypertensive medication.^2^ The regular practice of exercise, mainly aerobic exercises, is highly recommended due to its hypotensive effect.^3^ Actually, a single session of exercise is already able to promote a sustained reduction of blood pressure, this phenomenon is called post-exercise hypotension (PEH).^4-6^

Kenney and Seals^6^ were the first to term the most accepted version of PEH as a phenomenon - It is the decrease of systolic and/or diastolic blood pressure after an acute session of exercise to below a control value followed by no clinical hypotensive symptom. PEH has been faced as a clinically relevant tool, mainly due to its known magnitude and for lasting many hours.^5^ In this context, a meta-analysis including 65 studies recently showed reductions of blood pressure averaging from 6/4 mmHg for systolic/diastolic after aerobic exercise session,^7^ while hour-to-hour analysis reported a decrease for 16 hours.^8^

Despite established, PEH presents a large variation in magnitude and duration across the literature, which suggest that many factors of influence and different mechanisms are involved in promoting PEH.^4^ Along this line, Casonatto et al.^9^ suggested that citrulline malate oral supplementation might favor a greater PEH in middle-age treated hypertensives. For this, the authors supplemented the subjects with citrulline malate in a randomized double-blinded study controlled by placebo. The supplementation of citrulline malate increases arginine plasma levels, which favors the augment of nitric oxide through the cycle of citrulline-nitric oxide.^10^ Thus, the authors suggest that greater levels of nitric oxide were responsible for a greater decrease in systemic vascular resistance and subsequently PEH. However, in healthy subjects, Halliwill et al.^11^ did not observe any influence on blood pressure, calf and forearm vascular resistance post-exercise after inhibiting systemic nitric oxide synthase. It is also important to highlight that citrulline malate did not promote hypotensive effect stand-alone, which suggests a greater effect only when it is associated to exercise. Such results bring an open field for future studies to investigate how citrulline malate and aerobic exercise can together promote a greater PEH and the mechanisms behind it.

Although reproducibility is good for PEH,^12^ subjects present not uniform blood pressure responses post-exercise. Such pattern has encouraged researchers to explore individual analysis as an additional approach to show their data and not only the statistical difference for group means.^13,14^ The authors of the study discussed in this Short Editorial also highlighted inter-individual analysis in which they categorized “responders” (i.e. who the blood pressure decrease post-exercise) and “non-responders” (i.e. who the blood pressure did not change or was increased post-exercise). This type of analysis allows even though is observed no mean differences, most of the subjects might present clinically relevant blood pressure decrease post-exercise, which occurred for some variables in the discussed study. However, it is not still totally settled which is the best approach to interpret inter-individual data, and researchers should be careful about assumptions and conclusions when introduce this analysis.

The best strategy is still to be matched to define a “responder” and a “non-responder”, and the debate remains whether it needs to be based on changes clinically relevant or representing a measure defined by a mathematical approach. Concerning the magnitude of PEH to determine a clinically relevant change for PEH is also not still determined; a quite acceptable option might be employing the error of blood pressure measurement to be overcame by exercise reducing blood pressure below these values.^15^ Nonetheless, few well-designed studies have adequately investigated the reproducibility of PEH to characterize a universal error measurement. Then, to calculate the error in each study would be the best approach, taking blood pressure measurements at rest in two different days considering the subjects and the same evaluator involved in the study.

Thus, the results presented by Casonatto et al.^9^ suggest a possible associated effect of citrulline malate oral supplementation in promoting greater PEH in hypertensives, and which mechanisms are involved in this response should be explored in the future. Another unsolved question was raised in this study; might oral supplementation with citrulline malate associated to aerobic exercise be a promising tool to promote other cardiovascular benefits, such as vascular function, in both, acute and chronic studies?

Regarding analysis to report data, studies investigating group mean data demonstrated the clinical implications for PEH, but inter-individual analysis may be a step forward in the comprehension of this phenomenon. Then, to identify whether and what are the clinical meanings for “responders” and “non-responders”.
